# A Tailored Lipid Supplement Restored Membrane Fatty Acid Composition and Ameliorates In Vitro Biological Features of Human Amniotic Epithelial Cells

**DOI:** 10.3390/jcm11051236

**Published:** 2022-02-24

**Authors:** Valeria Pizzuti, Provvidenza Maria Abruzzo, Alexandros Chatgilialoglu, Silvia Zia, Pasquale Marrazzo, Giovannamaria Petrocelli, Chiara Zannini, Cosetta Marchionni, Paola Poggi, Giuliana Simonazzi, Silvia Canaider, Francesco Alviano, Federica Facchin, Laura Bonsi

**Affiliations:** 1 Unit of Histology, Embryology and Applied Biology, Department of Experimental, Diagnostic and Specialty Medicine, University of Bologna, 40126 Bologna, Italy; valeria.pizzuti3@unibo.it (V.P.); provvidenza.abruzzo2@unibo.it (P.M.A.); pasquale.marrazzo2@unibo.it (P.M.); giovannam.petrocell2@unibo.it (G.P.); cosetta.marchionni@unibo.it (C.M.); silvia.canaider@unibo.it (S.C.); federica.facchin2@unibo.it (F.F.); laura.bonsi@unibo.it (L.B.); 2 Unit of Nephrology, Dialysis and Renal Transplant, Department of Experimental, Diagnostic and Specialty Medicine, St. Orsola-Malpighi University Hospital, 40138 Bologna, Italy; 3 Remembrane S.r.l., 40026 Imola, Italy; alex.chatgilialoglu@remembrane.com (A.C.); paola.poggi@remembrane.com (P.P.); 4 Stem Sel S.r.l., 40127 Bologna, Italy; silvia.zia@stemsel.it; 5 National Laboratory of Molecular Biology and Stem Cell Bioengineering of the National Institute of Biostructures and Biosystems (NIBB)-ELDOR Lab, at the Innovation Accelerator, CNR, Via Piero Gobetti 101, 40129 Bologna, Italy; chiara.zannini5@unibo.it; 6 Obstetric Unit, Department of Medical and Surgical Sciences, Policlinico St. Orsola-Malpighi, University of Bologna, 40126 Bologna, Italy; giuliana.simonazzi@unibo.it

**Keywords:** stem cells, human Amniotic Epithelial Cells (hAECs), membrane fatty acid composition, lipid supplementation, immunomodulatory capacity

## Abstract

Cell culture conditions influence several biological and biochemical features of stem cells (SCs), including the membrane lipid profile, thus limiting the use of SCs for cell therapy approaches. The present study aims to investigate whether the in vitro culture may alter the membrane fatty acid signature of human Amniotic Epithelial Cells (hAECs). The analysis of the membrane fatty acid composition of hAECs cultured in basal medium showed a loss in polyunsaturated fatty acids (PUFA), in particular in omega-6 (ω-6) content, compared to freshly isolated hAECs. The addition to the basal culture medium of a chemically defined and animal-free tailored lipid supplement, namely Refeed^®^, partially restored the membrane fatty acid signature of hAECs. Although the amelioration of the membrane composition did not prolong hAECs culture lifespan, Refeed^®^ influenced cell morphology, counteracted the onset of senescence, and increased the migratory capacity as well as the ability of hAECs to inhibit Peripheral Blood Mononuclear Cell (PBMC) proliferation. This study provides new information on hAEC features during culture passages and demonstrates that the maintenance of the membrane fatty acid signature preserved higher cell quality during in vitro expansion, suggesting the use of lipid supplementation for SC expansion in cell-based therapies.

## 1. Introduction

Lipid metabolism influences stem cell behavior by affecting cell proliferation and differentiation ability [1] (p. 115). Lipids, and particularly fatty acids (FA), are the main components of biological membranes and exert an essential role in membrane structure maintenance, permeability, flexibility, and fluidity, among many others [2] (pp. 720–731). FA are involved in several signaling pathways and signal propagation [3] (p. 432), in energy storage and consumption [4] (pp. 151–162), in the sustainment of organelle structure and function, and in cell homeostasis [5] (p. 2167). Among FA, polyunsaturated fatty acids (PUFA), which include omega-3 (ω-3) and omega-6 (ω-6) classes, are considered essential fatty acids. Mammals are not able to synthesize PUFA precursors, linoleic (LA, ω-6), and alpha-linolenic (ALA, ω-3) acids, and they take up these essential FA from exogenous sources [6] (pp. 135–144) [7] (pp. 401–421).

It has been reported that both stem and primary cells modify their membrane fatty acid signature during cell culture, usually showing an increase in monounsaturated fatty acids (MUFA) and a decrease in PUFA content in comparison to uncultured cells [8,9] (pp. 1–11). The alteration of PUFA content affects paracrine features [10] (pp. 3225–3233), immune response [11] (p. 2142), and differentiation capacity of stem cells (SCs) [12] (pp. 411–417). The changes in membrane FA composition are caused by the inability of SCs to synthetize PUFA and by a limited access to this lipid family in the cell culture environment [13] (p. 101017). Accordingly, fetal bovine serum (FBS), the exogenous source of lipids commonly used in culture media, does not provide an adequate lipid supply both quantitatively and qualitatively [13] (p. 101017). Moreover, serum contains xenogeneic components that enhance the risk of immune reaction in transplanted patients [14] and exhibits adverse effects on differentiation capacity and proliferative activity of SCs [15] (p. 27). Therefore, to prevent cellular alterations and promote SC expansion, researchers have focused on the development of optimized culture systems that mimic the physiological environment, where SCs are able to proliferate and maintain their self-renewal ability [16] (pp. 552–569).

Fetal tissues, such as placenta and umbilical cord, and amniotic fluid constitute an interesting source of perinatal SCs, with several advantages, including high availability, easy cell collection, and absence of ethical concerns [17,18]. Perinatal cells show differentiation ability [19] (pp. 2–10), immunomodulatory capacity, anti-inflammatory, and anti-fibrotic activity [20] (pp. 53–63).

In a previous study, our research group analyzed the membrane fatty acid lipid signature and the influence of a chemically defined lipid supplement, namely Refeed^®^, on cultured mesenchymal/stromal stem cells (MSCs) derived from human fetal membranes (hFM-MSCs) [9] (pp. 1–11). hFM-MSCs cultured in basal medium modified their membrane fatty acid signature, showing a decrease in PUFA and an increase in MUFA content [9] (pp. 1–11), [14]. The addition of Refeed^®^ to the culture medium partially restored the membrane fatty acid signature and improved several characteristics of cultured hFM-MSCs, such as proliferative and immunomodulatory activity, as well as angiogenic differentiation capacity [9] (pp. 1–11). In a second study, we further demonstrated how Refeed^®^ supplementation affected hFM-MSC paracrine features by impacting intracellular vesicle trafficking, exosome production, and secretive functions [21] (pp. 55–69). human Amniotic Epithelial Cells (hAECs) represent another cell population derived from placenta; in particular, hAECs derive from the amniotic membrane [17] and originate from pluripotent epiblast cells. hAECs express typical pluripotency markers, such as octamer-binding protein 4 (OCT4), SRY-related HMG-box gene 2 (SOX2), and Nanog [22] (pp. 1549–1559). Moreover, these perinatal cells express several stem cell surface markers, such as stage-specific embryonic antigen-4 (SSEA-4) and SSEA-3, along with tumor rejection antigen 1–60 (TRA1-60) and TRA1- 81, which are known to be expressed in human embryonic stem cells (hESCs) [23] (pp. 329–337)**.** Due to their stemness features, hAECs can differentiate toward all three germ layers [24] (pp. 139–145); it has been demonstrated that hAECs are able to differentiate into insulin-secreting pancreatic β-islet-like cells [25] (pp. 390–402), hepatic-like cells [26] (pp. 1719–1729), and in surfactant-producing alveolar epithelial cells [27] (pp. 643–651), among others. Furthermore, hAECs are considered a safe source for cell therapies because they do not cause teratomas upon transplantation [24] (pp. 139–145), and they show migration ability, which allows them to migrate towards inflamed and damaged tissue sites [28] (pp. 700–709). Finally, hAECs exhibit immunomodulatory [29] (pp. 31–40) [30], anti-fibrotic and anti-inflammatory features [31] (pp. 404–409).

The clinical use of hAECs is hampered by their low expansion ability during in vitro culture [32,33] (pp. 955–968). To enhance their proliferative potential, hAECs have been cultured with exogenous epithelial growth factor (EGF) [34,35] (pp. 701–704, pp. 220–227); however, their prolonged in vitro maintenance has been associated with changes in their phenotypical features and in the expression of surface markers [33] (pp. 955–968). Still, the differentiation potential and immunomodulatory capacity of expanded hAECs are not completely understood.

The aim of this study was to investigate whether the in vitro culture may alter the membrane fatty acid signature of human Amniotic Epithelial Cells (hAECs). Compared to freshly isolated cells, cultured hAECs brought an increase in MUFA and strong alterations in PUFA classes. Then, we evaluated whether the use of the lipid supplement Refeed^®^, already tested in our previous studies [9,21] (pp. 1–11, pp. 55–69), may restore hAEC membrane fatty acid signature and influence hAEC biological properties, such as proliferation, migration, and immunomodulation ability. The Refeed^®^ supplement partially restored hAEC membrane fatty acid signature. Compared to untreated cells, hAECs treated with Refeed^®^ increased their migratory capacity, improved their ability to reduce the proliferation of Peripheral Blood Mononuclear Cells (PBMCs), and delayed the onset of senescence. These data provided new information about hAEC features during cell culture and suggested that the optimization of culture conditions by the use of a tailored lipid supplement is able to preserve hAEC properties and improve the maintenance and the expansion process of cultured cells.

## 2. Materials and Methods

### 2.1. Ethics Statement

This study was approved by the Local Ethical Committee (IRCCS St. Orsola-Malpighi University Hospital Ethical Committee, protocol n° 2481/2017, ref n° 68/2017/U/Tess). Placentas were obtained from healthy donor mothers undergoing elective caesarean section at term (37–40 weeks) after written informed consent. Tissues were maintained under sterile conditions until cell isolation.

### 2.2. Isolation of Human Amniotic Epithelial Cells (hAECs)

Fetal membranes were washed with ice-cold phosphate-buffered saline (PBS, Corning, NY, USA) with 1% penicillin-streptomycin solution (10,000 U/mL penicillin, 10,000 U/mL Streptomycin, Corning, Steuben County, NY, USA). Amniotic membrane was mechanically peeled off the underlying chorion layer; to remove any blood clots, tissues were incubated for 10 min at room temperature with PBS/ ethylene diaminetetraacetic acid (EDTA) 0.5 mM. The amniotic membrane was then minced into small pieces (4 cm^2^ approximately) and digested twice for 30 min at 37 °C using Trypsin-EDTA 0.25% (Corning, Steuben County, NY, USA) with gentle shaking. For both digestion steps, Trypsin was inactivated with fetal bovine serum (FBS, Gibco, Life Technologies, Carlsbad, CA, USA), and the cell suspension was centrifuged for 10 min at 390 g. The cell pellet was resuspended in basal culture medium, Dulbecco’s Modified Eagle’s Medium-high glucose (DMEM H., Corning, Steuben County, NY, USA) containing 10% FBS, 1% penicillin-streptomycin solution, and EGF, 10 ng/mL (Sigma-Aldrich, St. Louis, MO, USA). Single cell suspension was counted and tested for viability using Erythrosin B (Sigma-Aldrich, St. Louis, MO, USA). Only samples with > 90% viability were used for further assays.

### 2.3. Immunophenotypic Analysis of hAECs

Immunophenotypic characterization of hAECs was assessed after isolation by flow cytometry. hAECs were fixed for 10 min at room temperature using Intraprep Kit (Beckman–Coulter Inc., Brea, CA, USA) and washed twice with PBS. Cells were incubated for 30 min at 4 °C with conjugated primary antibodies (1 μg/mL) specific for epithelial (anti-panCytokeratin (Pan-Ck)-PE, Santa Cruz Biotechnology, Santa Cruz, CA, USA), mesenchymal (anti-CD44-FITC, anti-CD73-PE, anti-CD90-PC5, anti-CD105-PE, Beckman-Coulter Inc., Brea, CA, USA), and hematopoietic (anti-CD34-FITC, anti-CD45-APC, Beckman-Coulter Inc., Brea, CA, USA) markers. For the analysis of Pan-Ck during culture passages, we used the Cytokeratin Pan Type I/II Antibody Cocktail (MA5-13156, Thermo Scientific, Waltham, MA, USA) and Alexa Fluor 488 (A11001, Thermo Scientific, Waltham, MA, USA) as secondary antibody. After incubation, cells were washed with PBS and analyzed using the FACS Navio FC (Beckman-Coulter Inc., Brea, CA, USA) cytometer and the Kaluza FC Analysis software (Beckman-Coulter Inc., Brea, CA, USA).

### 2.4. hAEC Membrane Isolation and Fatty Acid Composition Analysis

Cell membranes of hAECs were collected from both freshly isolated (ISO) and cultured cells from P0 to P4. The purification of cell membranes was performed as previously described [9] (pp. 1–11). Briefly, cell membrane lipids were extracted with CHCl_3_/MeOH (2:1 *v*/*v*) and then incubated with 0.5 M KOH in methanol for 10 min at room temperature; thus, trans-esterifying fatty acids linked by ester bonds to methanol to form fatty acid methyl esters (FAMEs). FAMEs were extracted with n-hexane and separated by gas chromatography in an Agilent 7820 A GC System (Agilent Technologies, Santa Clara, CA, USA) fitted with a 60 m × 0.32-mm DB23 capillary column, film thickness 0.25 μm, and a flame ionization detector (FID). Helium was used as a carrier gas at 2.54 mL/min, and the split injector was used with a split ratio of 10:1. Injector temperature was 250 °C and detector temperature was 260 °C. The column oven temperature was maintained at 50 °C for 2 min after sample injection and was programmed for the following temperature gradient: 10 °C/min from 50 °C to 180 °C, 3 °C/min from 180 °C to 200 °C and holding at 200 °C for 6 min. The separation was recorded with G6714 AA SW EZChrom Elite Compact (Agilent Technologies, Santa Clara, CA, USA). FAMEs were identified by comparison with standards purchased from NuCheckPrep Inc. (Elysian, MN, USA). FAMEs are expressed in weight %, based upon the contribution of the peak area of each FAME in the chromatogram. To take into account the different signal of the detector for different molecules, a correction factor was applied to the experimental data coming from the integration of the chromatograms. The total of the peaks analyzed for each chromatographic run was 100.

### 2.5. Refeed^®^ Supplement

Refeed^®^ supplement (Remembrane Srl, Imola, Italy) is a completely defined combination of non-animal-derived lipids and antioxidants solubilized in 1 mL of ethanol. One milliliter of Refeed^®^ was diluted in 500 mL of complete cell growth medium, with the resulting ethanol concentration being <1% (*v*/*v*) in the final medium. Based on the similarity between the membrane fatty acid signature of hFM-MSCs and hAECs, in this work, we used the same Refeed^®^ formulation that has been used in our previous studies on hFM-MSCs [9,21] (pp. 1–11; 55–69).

### 2.6. hAEC Culture and Refeed^®^ Lipid Supplementation

After isolation, cells were seeded in basal culture medium without (Ctrl) or with lipid supplementation (Refeed^®^) at a density of 100,000 cells/cm^2^. At this step, half of the full dose (1:500) of Refeed^®^ was added to the basal culture medium to promote cell adaptation to the supplement. When hAECs reached confluence (P0), cells were harvested and seeded at 30,000 cells/cm^2^ (P1). From P1 to P4, cell density was maintained, and lipid supplement was added to the basal culture medium at the full dose. Culture medium with or without Refeed^®^ was changed every two days. Assays were performed from P0 to P4 depending on the purpose of the analysis.

### 2.7. hAEC Viability and Proliferation Analysis

To assess the effect of Refeed^®^ on cell viability, we counted the number of live and dead cells using a dye exclusion test. This assay is based on the principle that only live cells possess intact cell membranes, which exclude certain dyes, such as Trypan blue, eosin, or propidium [36]. We used the Erythrosin B, a red dye that was proven to be accurate as Trypan blue in cell counting [37] (pp. 8–12). Briefly, at each passage (P0–P4), untreated and Refeed^®^-treated hAECs were detached using trypsin-EDTA, and the cell suspension was mixed with Erythrosine B (0.2% in PBS). Not-stained viable cells and red-stained dead cells were counted using the Neubauer hemocytometer (BRAND GmbH, Wertheim, Baden-Wurttemberg, Germany) under a light microscope. Cell viability was obtained by calculating the percentage of living cells and the percentage of dead cells compared to the total number of cells.

The proliferative capacity of hAECs, cultured with or without Refeed^®^ after each passage (P0–P4), was assessed as Cumulative Population Doubling (CPD) by applying the following formula: [log10(NH)-log10(N1)]/log10(2)], where NH is the number of harvest cells, and N1 is the number of plated cells. Moreover, the expression of Ki67 was evaluated by immunofluorescence described below.

### 2.8. Immunofluorescence Analysis of hAECs

For every passage (P1–P4), hAECs were seeded onto glass coverslips and cultured in basal medium with or without Refeed^®^ supplementation. Cells were fixed with 10% formalin at room temperature, washed with PBS, and then permeabilized by adding PBS 0.1% Triton (Triton X-100, Sigma-Aldrich, Co., St. Louis, MO, USA) for 10 min. hAECs were incubated for 30 min with a blocking solution containing PBS 1% bovine serum albumin (BSA, Sigma-Aldrich, St. Louis, MO, USA) and then incubated overnight at 4 °C with the primary antibodies mouse anti-Pan-Ck (1:200, #sc-8018 Santa Cruz Biotechnology, Santa Cruz, CA, USA) and rabbit anti-Ki67 (1:250, #ab16667, Abcam, Cambridge, UK) diluted in blocking solution. Secondary antibodies anti-mouse Cy3 (1:250, #C2181, Sigma-Aldrich, St. Louis, MO, USA) and anti-rabbit Alexa Fluor Plus 594 (1:500, #A32754, Thermo Fisher Scientific, Waltham, MA, USA) were used for 1 h incubation at room temperature. After three washes with PBS, coverslips were mounted using the Prolong Gold Antifade Mountant with DAPI (Thermo Fisher Scientific, Monza, Italy). Stained cells were observed using Nikon Inverted Microscope (Nikon Instruments, Tokyo, Japan), and images were acquired with a Digital Sight camera DS-03 using the imaging software NIS-Elements (Nikon Corporation, Tokyo, Japan). To quantify the expression of Ki67, ten different fields for each condition were acquired, and the number of red-stained nuclei was counted and compared to total number of cells. Data are expressed as mean ± standard deviation (SD).

### 2.9. Morphological and Dimensional Analysis of hAECs

To investigate the effect of Refeed^®^ on cell morphology along passages (P0–P4), hAECs were monitored using a Leica Labovert FS inverted Microscope (Leica Microsystems, Wetzlar, Germany). When cells reached confluence, 10 images of the cell monolayer for each condition (Ctrl and Refeed^®^) were acquired using a Leica MC170 HD digital camera (Leica Microsystems, Wetzlar, Germany). The acquired images were used to calculate the cell area of hAECs using ImageJ software [38] (pp. 671–675). For each culture passage, results are reported as the mean of cell area (µm^2^) ± SD for both hAEC culture conditions.

### 2.10. Celector^®^ Technology: Fractionation Principle, Procedure, and Optical Analysis

Cell separation was obtained in a capillary device where cell suspensions were eluted through a laminar flow of physiological buffer. hAECs (300,000 cells) were injected at a flow rate of 1 mL/min into the system; cells reached a specific position across the channel thickness during transportation due to the combined action of gravity, acting perpendicularly to the flow and opposing lift forces that depend on the morphological features of the sample. Cells at a specific position in the channel acquired well-defined velocities, and therefore, they elute at a specific time [39] (pp. 9081–9087). The in-flow injection maintained the native properties and allowed a high sample recovery. The fractionation procedure involved at first the decontamination of the fractionation system by flushing with cleaning solution at 1 mL/min flow rate. Next, the system was washed copiously with sterile, demineralized water at the same flow rate. Although the Non-Equilibrium, Earth Gravity-Assisted Dynamic Fractionation (NEEGA-DF) method is optimized to prevent contact between cells and fractionation device, to block non-specific interaction sites on the plastic walls, the fractionation system was flushed at 0.5 mL/min with a sterile coating solution. Finally, it was filled with a sterile mobile phase. All solutions were provided by Stem Sel S.r.l., Italy. The instrument is placed under a laminar flow hood to maintain the sterility of the collected cells. Eluted hAECs were monitored using a micro-camera detector (MER-U3 camera, DAHENG IMAGING, Beijing, China) placed at the outlet of the fractionation channel. The imaging software (Celector^®^ Optics, Stem Sel S.r.l., Italy) recorded all frames of analysis, which were post processed to obtain information about dimension of single cells, as a function of time, population heterogeneity, and composition of possible sub-populations. Area of all eluted cells was visualized as a curve that represents average cell area as a function of time using the dedicated data processing software (Stem Sel Analyzer) and as graph bars for a selected time interval (cell fraction).

### 2.11. Senescence Analysis of hAECs by SA-β-Galactosidase Assay

The Senescence-Associated β-Galactosidase assay (SA-β-Gal) was performed on hAECs cultured from P1 to P4. Cells were seeded in 24-well culture plates (5000 cells/cm^2^) and cultured in basal medium with or without Refeed^®^. The SA-β-Gal assay was performed using a commercial kit (Cell Signaling Technology, Danvers, MA, USA) following the manufactured instructions. The staining reaction was stopped after six hours; images of stained hAECs were acquired using Leica Labovert FS inverted Microscope (Leica Microsystems, Wetzlar, Germany) with Leica MC170 HD digital camera (Leica Microsystems, Wetzlar, Germany), and plates were stored at 4 °C in 70% glycerol. The number of blue-stained SA-β-Gal-positive cells in 10 random fields of the wells was counted; data were analyzed using GraphPad Prism 7.04 software. Results are represented as the percentage of blue-stained cells (SA-β-Gal positive cells) ± SD at each passage for both conditions.

### 2.12. RNA Extraction and cDNA Synthesis

RNA extraction and cDNA synthesis were performed as previously described in detail [40]. Briefly, hAECs were seeded in T25 flasks at the density of 30,000 cells/cm^2^ and cultured in basal medium with or without Refeed^®^ supplementation. Untreated and Refeed^®^-treated cells were collected at P1, P3, and P4, and total RNA was extracted using the RNeasy mini kit (QIAGEN, Valencia, CA, USA) following the manufacturer’s instructions. The genomic DNA contamination was removed by digestion with RNase-free deoxyribonuclease I (RNase-free DNase set, QIAGEN, Valencia, CA, USA). The evaluation of RNA quality was performed according to Bolotta et al. [41]. RNA concentration was assessed using the NanoDrop^®^ 1000 Spectophotometer (Thermo Fisher Scientific, Waltham, MA, USA). The iScript cDNA Synthesis Kit (Bio-Rad Laboratories, Hercules, CA, USA) was used to reverse transcribe the RNA according to the manufacturer’s instructions.

### 2.13. Real-Time PCR

Real-time PCR (qPCR) was performed in a Bio-Rad CFX96 real-time thermal cycler (Bio-Rad Laboratories, Hercules, CA, USA) as previously described [40]. Briefly, for each condition, 25 ng of cDNA were amplified using the SsoAdvanced Universal SYBR Green Supermix (Bio-Rad Laboratories, Hercules, CA, USA) in technical triplicate. Primers for CDKN1A (p21^WAF1^, p21) and CDKN2A (p16^INK4A^, p16) (20×, unique assay ID: qHsaCID0014498 and qHsaCED0056722, respectively, Bio-Rad Laboratories, Hercules, CA, USA) were designed by Bio-Rad and used following the manufacturer’s instructions. Data were analyzed using the software CFX Manager (Bio-Rad Laboratories, Hercules, CA, USA) and the 2^−ΔΔCt^ method [42] (pp. 402–408). The TATA box binding protein (TBP) and glyceraldehyde-3-phosphate dehydrogenase (GAPDH) (20×, unique assay ID: qHsaCID0007122, qHsaCED0038674, Bio-Rad Laboratories, Hercules, CA, USA) were used as reference genes. For each gene, the normalized expression value of untreated hAECs at P1 was set to 1, and the other gene expression data were reported to that sample. Data are expressed as fold change  ± SD.

### 2.14. Analysis of hAEC Migratory Potential by Scratch Wound Assay

Scratch assay was performed on hAECs from P1 to P4 in both basal medium and Refeed^®^ supplemented one. Cells, seeded at 60,000 cells/cm^2^ in a 24-well culture plate, were allowed to grow for 24 h to obtain a confluent monolayer. The scratch was made using plastic sterile tips, and then, wells were washed with PBS to remove detached cells. Fresh culture medium with or without Refeed^®^ was added to the wells. Scratched monolayers were monitored, and images were acquired using an optical microscope Leica Labovert FS inverted Microscope (Leica Microsystems, Wetzlar, Germany) with a Leica MC170 HD digital camera (Leica Microsystems, Wetzlar, Germany) at regular interval times until their complete closure. Scratch areas were measured with the NIH ImageJ program [38] (pp. 671–675), and the percentage of scratch closure was calculated using GraphPad Prism software. The results are represented as the percentage of uncovered area ± SD. The scratch area at T0 was set as 100% for both culture conditions.

### 2.15. Isolation of Peripheral Blood Mononuclear Cells (PBMCs)

PBMCs were obtained from the blood of healthy donors according to the protocol approved by the Ethics Committee. PBMCs were isolated by density gradient centrifugation with Histopaque^®^-1077 (Sigma-Aldrich, St. Louis, MO, USA) and counted with methyl violet (Sigma-Aldrich, St. Louis, MO, USA) to exclude red blood cells; cells were frozen at –80 °C in FBS with 10% of dimethyl sulfoxide (Sigma-Aldrich, St. Louis, MO, USA) and used within a month. To exclude donor variability, PBMCs from the same donor were used in all experiments.

### 2.16. Analysis of BrdU Incorporation in PBMCs Co-Cultured with hAECs

To investigate how Refeed^®^ affected the ability of hAECs to inhibit the proliferation of immune cells, hAECs were co-cultured with phytohemagglutinin (PHA)-activated PBMCs from P1 to P4. Briefly, hAECs were plated in 96-well culture plates at 30,000 cells/cm^2^ in basal culture medium with or without Refeed^®^ for 24 h. Then, PBMCs were thawed and washed twice with warm RPMI 1640 (Roswell Park Memorial Institute 1640, Corning, Steuben County, NY, USA) containing 10% FBS. Cells were counted with methyl violet and with Erythrosin B to test cell viability after thawing, which was higher than 90%. PBMCs, seeded at a density of 200,000 PBMCs/well on hAECs in 200 µL/well of fresh basal RPMI containing 10% FBS and EGF 10 ng/mL, were activated with 5 µg/mL of PHA (Sigma-Aldrich, St. Louis, MO, USA) and cultured with (PBMCs + PHA + Refeed^®^ (hAECs)) or without Refeed^®^ (PBMCs + PHA (hAECs)). Positive and negative controls were included; in particular, positive samples were obtained by culturing PBMCs with PHA both in basal (PBMCs + PHA) and in Refeed^®^ supplemented medium (PBMCs + PHA + Refeed^®^). PBMCs, without PHA stimulation, cultured with (PBMCs − PHA + Refeed^®^) or without Refeed^®^ (PBMCs – PHA) were included as negative controls. After 72 h of incubation at 37 °C and 5% CO_2_, PBMCs were collected, diluted 1:3 with RPMI medium + 10% FBS, and seeded in a new 96-well plate to assess cell proliferation by measuring the levels of bromodeoxyurdine (BrdU) incorporation using the Cell Proliferation ELISA BrdU colorimetric kit (Roche, Basel, Switzerland) according to the manufacturer’s instructions. Data show the percentage of BrdU incorporation of PBMCs co-cultured with hAECs with or without Refeed^®^, with respect to positive controls, PBMCs + PHA + Refeed^®^ and PBMCs + PHA, respectively, both set at 100%.

### 2.17. Statistical Analysis

All the experiments were performed at least on three human samples in technical triplicate. Data are presented as mean ± standard deviation (SD) and were analyzed by two-way ANOVA or *t*-test using Graph Pad Prism 7.04 software (San Diego, CA, USA). The significance threshold was *p* < 0.05.

## 3. Results

### 3.1. Immunophenotypic Characterization of Isolated hAECs

**Figure 1:**
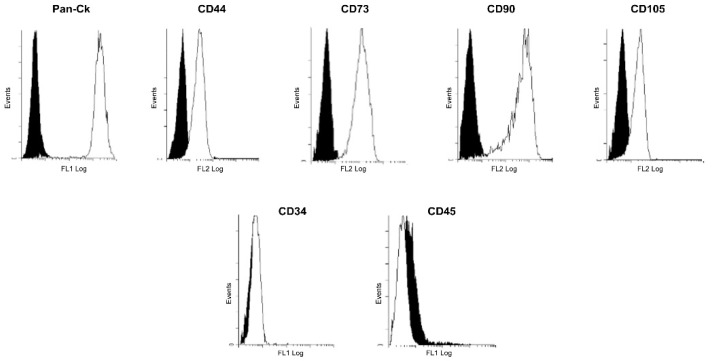
Immunophenotype of isolated human Amniotic Epithelial Cells (hAECs). Flow cytometry analysis of epithelial (Pan-Cytokeratin, Pan-Ck), mesenchymal (CD44, CD73, CD90, CD105), and hematopoietic (CD34, CD45) markers. Data are representative of three independent experiments obtained from three human samples. Black and white histograms represent the unstained controls and the specific cell markers, respectively.

To characterize the immunophenotypic profile of freshly isolated hAECs, epithelial, mesenchymal, and hematopoietic markers were evaluated by flow cytometry. As shown in Figure 1, hAECs expressed high levels of Pan-Cytokeratin (Pan-Ck), confirming their epithelial phenotype. Moreover, hAECs were positive for mesenchymal surface markers CD44, CD73, CD90, and CD105 and negative for hematopoietic markers CD34 and CD45 (Figure 1). These results are in line with previous studies [25,33] (pp. 390–402, 955–968).

### 3.2. Refeed^®^ Supplementation Restored the Membrane Fatty Acid Signature of hAECs during Cell Culture

**Figure 2:**
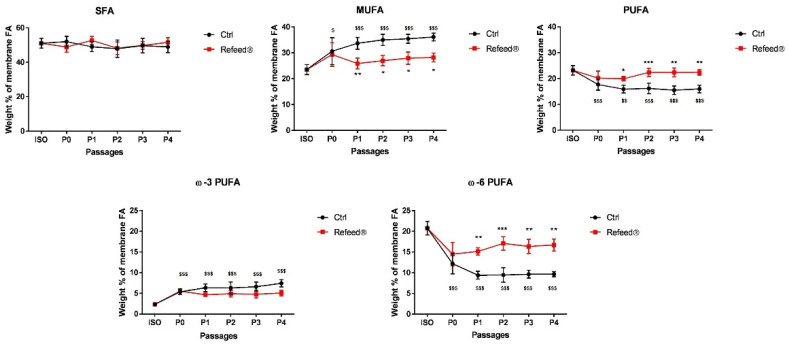
Membrane fatty acid profile of human Amniotic Epithelial Cells (hAECs) cultured with and without Refeed^®^ supplementation. Membrane fatty acid profile of freshly isolated hAECs (ISO), and of hAECs cultured in basal medium without (Ctrl) or with Refeed^®^ at passages P0, P1, P2, P3, and P4. Data are expressed in weight % of total membrane fatty acids and presented as mean ± standard deviation (SD). * indicates the statistical significance between Ctrl and Refeed^®^ at P0, P1, P2, P3, and P4 (* *p* < 0.05, ** *p* < 0.01, *** *p* < 0.001). ^$^ indicates the statistical significance between ISO and Ctrl at P0, P1, P2, P3, and P4 (^$^ *p* < 0.05, ^$$^ *p* < 0.01, ^$$$^ *p* < 0.001). SFA, saturated fatty acids; MUFA, monounsaturated fatty acids; PUFA, polyunsaturated fatty acids; ω-3 PUFA, omega-3 fatty acids; ω-6 PUFA, omega-6 fatty acids. The experiment was performed on six human samples in technical triplicate.

The membrane fatty acid composition was analyzed in both freshly isolated hAECs (ISO) and in hAECs cultured in basal medium from P0 to P4. As shown in Figure 2, the membrane fatty acid signature of ISO hAECs was characterized by saturated fatty acids (SFA) (51.04 ± 2.88%), MUFA (23.58 ± 1.9%) and PUFA (23.19 ± 1.82%). Among PUFA, ω-6 FA were the most abundant FA (20.76 ± 1.64%), while ω-3 FA were present in a small percentage (2.35 ± 0.47%).

When hAECs were cultured in basal medium from P0 to P4, significant changes in their membrane fatty acid signature were observed. As reported in Figure 2, at P0 hAECs showed a decrease in PUFA content (17.68 ± 2.21%) counterbalanced by an increase in MUFA content (30.60 ± 5.20%), while SFA content remained stable. Among PUFA, a slight increase in ω-3 FA (5.37 ± 0.68%) and a strong decrease in ω-6 FA (12.19 ± 2.46%) was observed. These changes were conserved during subsequent in vitro passages (P1-P4).

The observed alterations were partially restored when the tailored lipid supplement Refeed^®^ was added to the basal medium (Figure 2). As previously reported [9] (pp. 1–11), Refeed^®^ supplement is constituted by a combination of chemically defined and animal-free lipids and antioxidants. Refeed^®^ supplementation lowered MUFA content by reducing the loss of PUFA in cultured cells; in particular, the percentage of ω-6 FA reached levels similar to those of ISO cells at passage P2 and remained stable at P3 and P4. Refeed^®^ treatment did not affect significantly ω-3 FA content. These results demonstrated that Refeed^®^ supplement partially counteracted the alterations of hAEC membrane system caused by the standard cell culture passaging.

### 3.3. Effect of Refeed^®^ Supplementation on hAEC Viability and Proliferation

**Figure 3:**
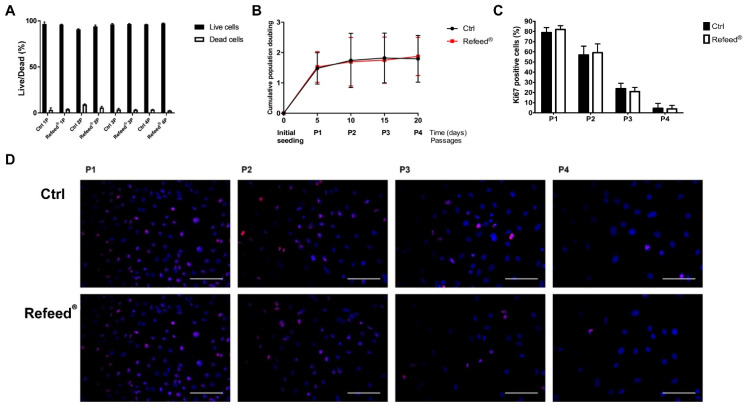
Analysis of viability and proliferation in human Amniotic Epithelial Cells (hAECs) cultured with and without Refeed^®^ supplementation. hAECs were cultured in basal medium (Ctrl) and in basal medium supplemented with Refeed^®^. (**A**) Percentage of living and dead hAECs during passages (P1–P4). (**B**) cumulative population doubling vs. passages (P0–P4) or days. (**C**) Representative images of Ki67 marker (nuclear red signal) during passages (P1–P4); nuclei were stained with DAPI (blue signal). Immunofluorescence images were acquired using the Nikon Inverted Microscope equipped with a Digital Sight camera DS-03. Scale bars: 100 μm. (**D**) Percentage of red-stained Ki67 positive cells from P1 to P4. Data are expressed as mean ± standard deviation (SD). The experiment was performed on three human samples in technical triplicate.

Cell viability was assessed by evaluating the percentage of living and dead cells from P1 to P4. As reported in Figure 3A, the percentage of dead cells was similar in both conditions, suggesting that Refeed^®^ did not affect cell viability. The analysis of the cumulative population doubling showed a decrease in the proliferation rate during culture, and no difference between untreated and treated cells was observed (Figure 3B). This result was confirmed by Ki67 expression, a nuclear protein widely associated with cell proliferation (Figure 3D). The immunofluorescence analysis showed a decrease in the percentage of Ki67-positive cells (nuclear red signal) during cell culture (Figure 3C), regardless culture conditions.

### 3.4. Expression of Pan-Cytokeratin in hAECs Cultured with or without Refeed^®^ Supplement

**Figure 4:**
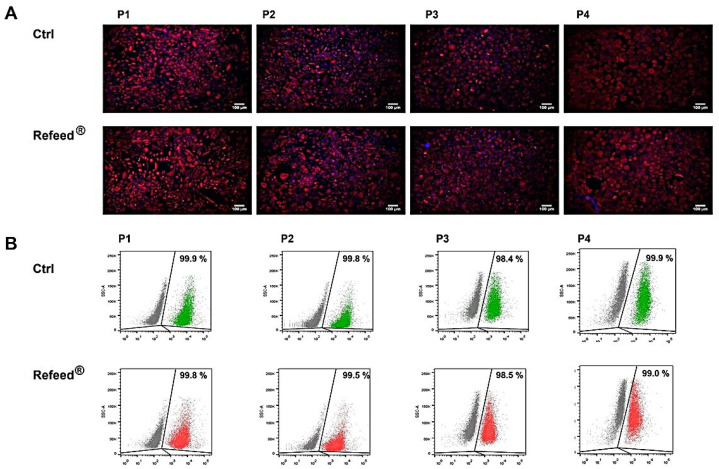
Expression of Pan-Cytokeratin (Pan-Ck) in human Amniotic Epithelial Cells (hAECs) cultured with and without Refeed^®^ supplementation. hAECs were cultured in basal medium (Ctrl) and in basal medium with Refeed^®^ supplement from passage 1 to passage 4 (P1–P4). (**A**) Immunofluorescence microscopy for the analysis of Pan-Ck marker (red signal); nuclei were stained with DAPI (blue signal). Images were acquired using the Nikon Inverted Microscope equipped with a Digital Sight camera DS-03. Scale bars: 100 μm. (**B**) Flow cytometry detection of Pan-Ck marker during passages (P1–P4). Representative images were chosen from three independent immunofluorescence and flow cytometry experiments.

In order to verify whether Refeed^®^ might affect the immunophenotype of cultured hAECs, the expression of the epithelial marker Pan-Ck was evaluated by immunofluorescence. As shown in Figure 4A, the expression of Pan-Ck in hAECs cultured with Refeed^®^ supplement was comparable to untreated cells (Ctrl) during all passages. The expression of Pan-Ck in cultured hAECs was further evaluated by flow cytometry analysis; data showed a positive expression of Pan-Ck in hAECs during culture (P1–P4), without significant differences between the two culture conditions (Figure 4B). Both immunofluorescence and cytometry results confirmed the maintenance of the epithelial phenotype during cell culture and demonstrated that Refeed^®^ treatment did not influence Pan-Ck expression.

### 3.5. Effect of Refeed^®^ Supplementation on hAEC Morphology

**Figure 5:**
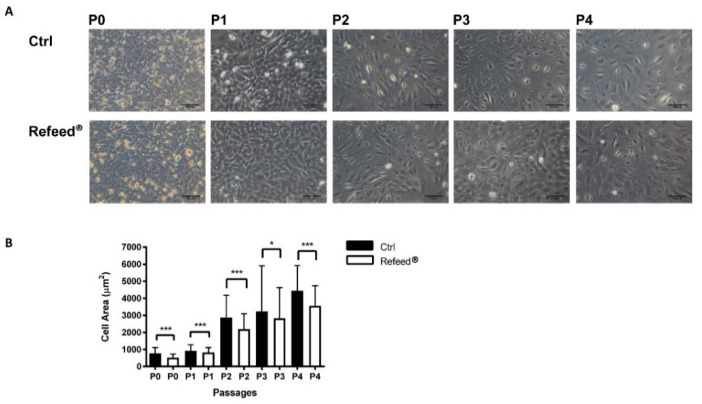
Morphological analysis of human Amniotic Epithelial Cells (hAECs) cultured with and without Refeed^®^ supplementation. (**A**) Representative images of hAECs cultured in basal medium without (Ctrl) or with Refeed^®^ were acquired from passages P0 to P4 using a Leica Labovert FS inverted Microscope equipped with a Leica MC170 HD digital camera. Scale bars = 100 µm. (**B**) Analysis of the hAEC area (µm^2^) during in vitro passages (P0–P4) in basal medium (Ctrl) and with Refeed^®^. Data are expressed as mean ± standard deviation (SD) (* *p* < 0.05, *** *p* < 0.001). The experiment was performed on three human samples in technical triplicate.

The morphology of hAECs cultured in both conditions was monitored from P0 to P4; a progressive increase in hAEC size was observed in both groups (Figure 5A). In particular, hAECs showed a strong change in cell dimension between P1 and P2. Interestingly, Refeed^®^ supplementation reduced the cell size in all investigated culture passages compared to Ctrl (Figure 5A). These observations were confirmed by the dimensional analysis (Figure 5B) obtained measuring cell area.

### 3.6. Effect of Refeed^®^ Supplementation on hAEC Dimension

**Figure 6:**
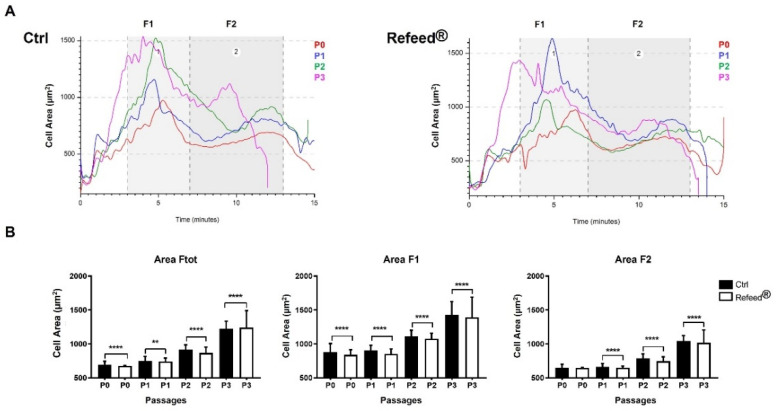
Analysis of human Amniotic Epithelial Cells (hAECs) area using Celector^®^ technology. (**A**) Dimensional profile of hAECs cultured in basal medium (Ctrl) and with Refeed^®^ supplement during passages P0–P3. Curves represent the cell area (µm^2^) vs. the time of analysis (minutes). Two sub-populations, F1 and F2, were underlined and further dimensional analysis were carried on following these time intervals. (**B**) Column graphs represent the cell area of hAECs for each passage in the whole population (F tot) and in subpopulations (F1 and F2) that were examined. Data are expressed as mean ± standard deviation (SD) (** *p* < 0.01, **** *p* < 0.0001). The experiment was performed on three human samples in technical triplicate.

Expanded hAECs were analyzed using Celector^®^, an instrument that exploits the NEEGA-DF method, to characterize and label-free separate cells based solely on their physical characteristics; bigger and denser cells exit the fractionation channel earlier than smaller and lighter cells. Freshly isolated hAECs were previously analyzed [43] (p. 782). In this work, we focused on the morphological fingerprint of hAECs to discern dimensional composition of cell population in the Refeed^®^ treated and untreated cells. Curves representing cell area versus time of analysis showed the presence of two sub-populations, one eluting in the first minutes (3–7 min, fraction 1, F1), characterized by bigger cells and small aggregates, followed by the second population (7–13 min) composed of smaller cells (fraction 2, F2) (Figure 6A). Moreover, Refeed^®^-treated hAECs were smaller than control cells at every passage in culture. The big change in cell dimension was found at P2, and was more evident at P3. When the two sub-populations, F1 and F2, were carefully analyzed, F1 cells always resulted larger than F2 cells regardless culture conditions (Figure 6B). Furthermore, a significant lower cell area was observed at every passage in both Refeed^®^-treated F1- and F2-cells compared to Ctrl. These results confirmed microscopic analysis.

### 3.7. Refeed^®^ Supplementation Reduced the Number of hAEC Senescent Cells

**Figure 7:**
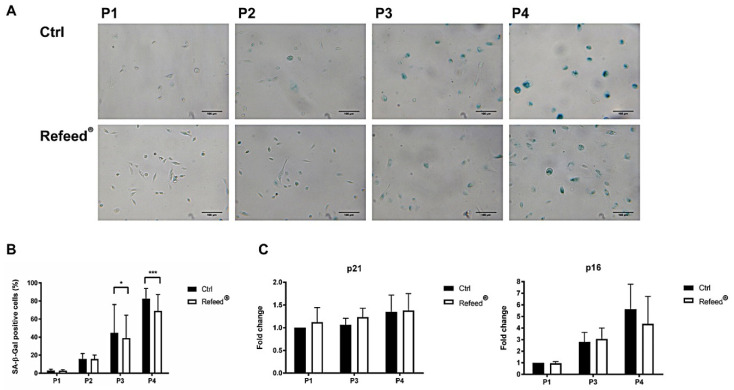
Senescence analysis of human Amniotic Epithelial Cells (hAECs) cultured with and without Refeed^®^ supplement. (**A**) Representative images of hAECs, cultured from passage 1 to 4 (P1–P4) in basal medium (Ctrl) and with Refeed^®^ supplement were acquired after SA-β-Gal staining. Images were obtained using the optical microscope Leica Labovert FS inverted Microscope equipped with a Leica MC170 HD digital camera. Scale bars = 100 µm; (**B**) the percentage of blue-stained SA-β-Gal-positive cells was calculated during in vitro cell culture. Results are represented as mean ± standard deviation (SD), (* *p* < 0.05, *** *p* < 0.001); (**C**) gene expression analysis of p16 and p21 in hAECs cultured without (Ctrl) or with Refeed^®^ at P1, P3, and P4. Data were normalized to two reference genes, TBP (TATA box binding protein) and glyceraldehyde-3-phosphate dehydrogenase (GAPDH); the normalized expression value of untreated hAECs at P1 was set to 1, and the other gene expression data were reported to that sample. Data are expressed as fold change  ± SD. The experiment was performed on three human samples in technical triplicate.

In order to analyze the effects of Refeed^®^ supplementation on cellular senescence, Senescence-Associated β-Galactosidase colorimetric assay (SA-β-Gal) was performed on hAECs cultured with or without Refeed^®^ from P1 to P4. Senescent hAECs were characterized by an intense blue staining (SA-β-Gal-positive cells), while non-senescent cells appeared unstained (SA-β-Gal-negative cells). Quantification showed an increase in cell senescence during culture passages, both in untreated and Refeed^®^-treated hAECs (Figure 7A,B). In particular, while the percentage of SA-β-Gal-positive cells at P1 and P2 was negligible, at P3 and P4, most of the hAECs were senescent. The addition of Refeed^®^ counteracted senescence phenomenon at late passages (P3-P4), with a statistical difference compared to Ctrl cells at P3 (44.78 ± 31.23% vs 38.84 ± 25.56%, *p* < 0.05) and at P4 (68.92 ± 18.32% vs 82.51 ± 11.29%, *p* < 0.01). To strengthen these data, we analyzed the gene expression of two cyclin-dependent kinase inhibitors, p16^INK4A^ (p16) and p21^WAF1^ (p21). We found an increase in p16 expression during cell culture in both control and Refeed^®^ treated-cells, while the expression of p21 remained unchanged (Figure 7C). Moreover, at P4 hAECs treated with Refeed^®^ showed a decreasing trend in p16 expression.

### 3.8. Effect of Refeed^®^ on the Migratory Capacity of hAECs

**Figure 8:**
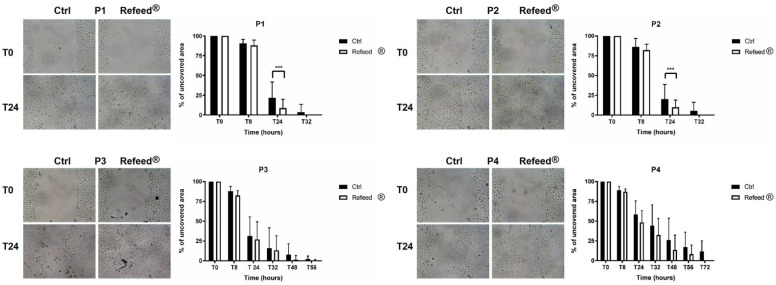
Migratory capacity of human Amniotic Epithelial Cells (hAECs) cultured with and without Refeed^®^ supplementation. For each passage (P1–P4), representative images of hAEC monolayer cultured with (Refeed^®^) or without (Ctrl) Refeed^®^ and the analysis of the scratch area are reported for both conditions. Images were acquired immediately after the scratch (T0) and 24 h after (T24), using the optical microscope Leica Labovert FS inverted Microscope equipped with a Leica MC170 HD digital camera. The percentage of the uncovered area of hAECs in basal medium (Ctrl) and with Refeed^®^ supplement was evaluated at the beginning of the assay (T0) and at different time points (T8, T24, T32, T48, T56, T72) until scratch was closed. The uncovered area at T0 is set to 100% for both conditions. Results are expressed as mean ± standard deviation (SD) (*** *p* < 0.001). The experiment was performed on three human samples in technical triplicate.

To assess the migratory capacity of hAECs cultured in basal or in supplemented Refeed^®^ medium, the scratch assay was performed from P1 to P4. As shown in Figure 8, the migratory capacity of hAECs decreased during passages in both culture conditions, and this observation was confirmed by the measurement of the scratch area. However, the migratory ability of hAECs was significantly influenced by the addition of Refeed^®^ to the culture medium, especially at the first stages of the culture. At P1 and P2, hAECs cultured with Refeed^®^ completely obliterated the scratch wound after 24 h, while at the same time, the scratch wound was still visible in Ctrl. At P3 and P4, although Refeed^®^ seemed to reduce the time for scratch wound closure, this effect was not significant compared to Ctrl hAECs. All together, these results suggested that although the migratory capacity of hAECs decreased during cell culture, Refeed^®^ supplementation lowered the time required for scratch wound closure.

### 3.9. Effect of Refeed^®^ on the Ability of hAECs to Inhibit PBMC Proliferation

**Figure 9:**
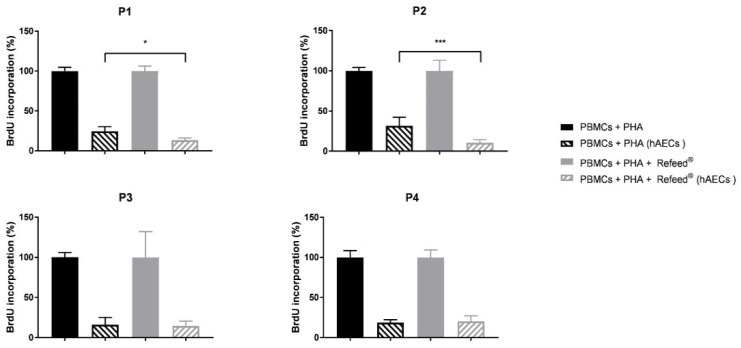
Percentage of bromodeoxyuridine (BrdU) incorporation in Peripheral Blood Mononuclear Cells (PBMCs) co-cultured with human Amniotic Epithelial Cells (hAECs), with and without Refeed^®^ supplementation. hAECs were co-cultured with phytohemagglutinin (PHA)-activated PBMCs for 72 h in basal medium (PBMCs + PHA (hAECs)) or in basal medium supplemented with Refeed^®^ (PBMCs + PHA + Refeed^®^ (hAECs)). PBMC proliferation was analyzed evaluating the percentage of BrdU incorporation. Positive controls were represented by activated PBMCs cultured in basal medium (PBMCs + PHA) and in basal medium supplemented with Refeed^®^ (PBMCs + PHA + Refeed^®^) without hAECs. Each percentage of BrdU incorporation of co-cultures was normalized with respect to their positive control, set at 100%. Results are expressed as mean of percentage of BrdU incorporation ± standard deviation (SD) (* *p* < 0.05, *** *p* < 0.001). The experiment was performed on three human samples in technical triplicate.

The effect of Refeed^®^ on the ability of hAECs to inhibit Peripheral Blood Mononuclear Cell (PBMCs) proliferation was evaluated by co-culturing hAECs with phytohemagglutinin (PHA)-activated PBMCs; the proliferative response of PBMCs was assessed measuring the percentage of bromodeoxyuridine (BrdU) incorporation. As shown in Figure 9, hAECs were able to inhibit PBMC proliferation in both culture conditions with respect to the positive control (PHA-activated PBMCs with or without Refeed^®^) during long-term in vitro expansion. The addition of Refeed^®^ to the basal medium significantly enhanced the ability of hAECs to inhibit PBMC proliferation during early in vitro passages (P1 and P2); On the contrary, at P3 and P4, no significant differences were observed between untreated and Refeed^®^-treated hAECs.

## 4. Discussion

In recent years, SCs have become a useful tool in cell-based therapy and regenerative medicine. Among SCs, epithelial cells isolated from amniotic membrane (hAECs) have been recently studied and characterized in virtue of their embryonic-like features and ability to differentiate into several cell types from all three germ layers [19,44] (pp. 2–10, p. 1304). Moreover, hAECs exhibit tolerogenic, immunomodulatory, migratory, and anti-inflammatory properties [18,29] (pp. 31–40), making these cells particularly attractive for clinical applications.

Unfortunately, hAECs show a low proliferative activity and a poor ability to maintain stem features during cell culture, thus limiting their potential use in cell therapeutic approaches [32]. It has been reported that SCs are particularly sensitive to the in vitro environment, which may influence stemness properties and differentiation abilities [45] (p. 9834).

In this study, we demonstrated for the first time the modification of hAEC membrane fatty acid signature during cell culture. The membrane fatty acid signature of freshly isolated hAECs, characterized by a high level of ω-6 FA, reflected the placenta-derived cells [9] (pp. 1–11). Cultured hAECs showed a significant loss in PUFA content and especially in ω-6 (Figure 2). These alterations, which occur mainly at the beginning of the in vitro culture (P0), originated both from the inability of hAECs to self-produce PUFA precursors [46] (pp. 31–50) and from the relatively low presence of ω-6 FA in the FBS added to the basal culture medium. The increase in MUFA content was in response to the loss of membrane fluidity originated by the drop of PUFA double bonds; by upregulating the activity of the Stearoyl-CoA-Desaturase, cells were able to partially counterbalance the drop in membrane fluidity through the synthesis of MUFA. The slight increase in ω-3 content was probably due to both the relatively high amount of ω-3 fatty acid supplied by the FBS and to the competition for the same enzymes by ω-6 and ω-3 fatty acid synthetic pathways [47,48] (pp. 77–80, pp. 22254–22266).

The in vitro establishment of an altered hAEC membrane fatty acid signature prompted us to add to the culture medium a tailored lipid supplement, Refeed^®^, to investigate its effect in counteracting these changes. Such supplement was previously tested on cultured hFM-MSCs [9] (pp. 1–11) and was developed according to the needs and the characteristics of the cell population of interest [9,49] (pp. 1–11, p. 165).

In our study, Refeed^®^ supplementation provided a mixture of different lipids that, by acting synergically, were able to partially restore the in vivo fatty acid profile of hAECs by lowering MUFA and raising PUFA content, ω-6 in particular (Figure 2). The addition of Refeed^®^ to the culture medium preserved hAEC viability and did not alter their natural epithelial phenotype (Figure 3A and Figure 4). Interestingly, the supplement led to an improvement of important biological properties of cultured hAECs. These effects could be explained by the Refeed^®^ ability to restore a physiological composition in the hAEC membrane system. As matter of fact, membrane FA regulate many important aspects of cell physiology, such as signaling and trafficking pathways, membrane fluidity, inflammation, and cell homeostasis [4,7] (pp. 151–162, pp. 401–421). In agreement to previous data [50], hAEC morphology changed during in vitro passages, showing an increase in cell dimension (Figure 5 and Figure 6); moreover, a reduction in hAEC proliferative capacity was observed (Figure 3B–D). Although Refeed^®^ did not influence hAEC proliferation, it delayed the increase in hAEC cell size without preventing it.

The increase in cell dimension and the decrease in cell proliferation are two of the main features of senescent cells, which appear enlarged, flattened, and with a multinucleated morphology [51,52] (pp. 56–80, pp. 729–740). Moreover, senescent cells are characterized by shortened telomeres, increased activity of SA-β-Gal, irreversible cell cycle arrest, and development of the complex Senescence-Associated Secretory Phenotype (SASP) [51] (pp. 56–80), [53,54]. In addition, senescence induces alteration in the expression of genes involved in cell cycle control, such as two cyclin-dependent kinase inhibitors p21 (p21^WAF1^) and p16 (p16^INK4a^). p21 and p16 are two key components of the two tumor-suppressor pathways (p53/p21 and p16/pRb), which are governed by the p53 and retinoblastoma (pRB) proteins, respectively [52,55] (pp. 729–740, pp. 75–95). The p53/p21 pathway is activated in response to DNA damage caused by telomere attrition and oxidative or oncogenic stress, while the p16/pRb pathway is mediated by stress stimuli, including overexpression of oncogenes, such as RAS and suboptimal culture conditions (lack of nutrients and growth factors) [52] (pp. 729–740). It has been demonstrated that oxidative stress (OS)-induced senescence in fetal membrane cells was associated with parturition at term [56] (pp. 1740–1751). However, the evaluation of senescence during hAEC in vitro culture remains unexplored. Senescence analysis of hAECs cultured with or without Refeed^®^ was assessed during cell passages. The percentage of senescent hAECs and the expression of p16 increased during passages in both culture conditions suggesting that the p16/pRb pathway was involved in our model and that a senescence phenotype was established during cell culture. However, the addition of Refeed^®^ was associated with a lower percentage of SA-β-Gal-positive cells and to a decreasing trend of p16 expression at late passages (Figure 7). These results could suggest that the restoration of the membrane fatty acid signature delays the progressive increase in hAEC senescence, probably due to an improvement of the cell oxidative metabolism. In fact, the maintenance of a physiological membrane fluidity improves oxygen diffusion through the cell membrane, leading to an increase in mitochondria efficiency and functionality [57] (pp. 181–187), ensuring a more effective control of the reactive oxygen species (ROS) production. It is known that ROS are associated with a senescent phenotype [58,59] (pp. 30–36). Moreover, recent evidences suggest that senescence is closely related to autophagy [60] (pp. 21485–21492); in particular, it was reported an anti-senescence role of autophagy in mesenchymal stem cells (MSCs) by clearing injured cytoplasmic organelles, such as mitochondria, and damaged molecules [61] (p. 276). Interestingly, it was observed that the supplementation of ω-6 PUFA induces autophagy in human epithelial cells as well as in *C. elegans*, leading in the latter case to an increase in its life span [62] (pp. 429–440). Since cell senescence negatively affects SC migration and homing capacity [63] (pp 1505–1519), scratch wound assay was performed in hAECs cultured with or without Refeed^®^. The migratory ability of hAECs decreased during in vitro expansion in both culture conditions although the addition of the lipid supplement was associated with an early closure of the scratch area in the first stages of the culture (P1 and P2) (Figure 8). Another important feature of hAECs is their immunosuppressive activity [18]; as previously reported [9,64] (pp. 1–11), this ability was assessed evaluating the proliferation of PHA-activated PBMC co-cultured with hAECs. hAECs have significantly reduced PBMC proliferation in both culture conditions during all culture passages (Figure 9), suggesting that the immunomodulatory potential of hAECs was not influenced by long-term maintenance despite the onset of a senescent phenotype. Moreover, we observed that at P1 and P2, Refeed^®^ supplementation enhanced the ability of hAECs to inhibit PBMC proliferation, suggesting that the maintenance of a physiological cell membrane composition positively affected the immunomodulatory activity of hAECs by modulating the paracrine features and regulating protein synthesis and folding as well as intracellular vesicle trafficking [21] (pp. 55–69). 

In conclusion, our study demonstrated that hAECs profoundly modified their membrane fatty acid signature during cell culture. The addition of a tailored lipid supplement to the basal culture medium partially restored the membrane fatty acid signature and resulted in an enhancement of the migratory capacity and of the immunosuppressive properties of hAECs as well as in a reduction of the number of senescent positive cells. These data suggest that the tuning of specific culture conditions is the way to improve the expansion process of cultured cells and to standardize cell culture protocols for Good Cell Culture Practice and cell therapy applications. As previously reported for hFM-MSCs [9] (pp. 1–11), hAECs also benefited from the addition of the same Refeed^®^ formulation at the same dose during in vitro maintenance. Such findings could be relevant in view of the setup of hAEC and hFM-MSC co-cultures and 3D cell models, which may represent intriguing future applications of placenta-derived SC.

## Data Availability

The datasets generated and/or analyzed in this study are not publicly available due to the know-how management police of Remembrane Srl, but they are available from the corresponding author from reasonable request.
